# Environmental, Lifestyle, and Medical Risk Factors Associated With Multiple Sclerosis: A Case-Control Study in Kermanshah, Iran

**DOI:** 10.34172/jrhs.11608

**Published:** 2026-02-21

**Authors:** Nazanin Razazian, Asma Aliahmadi, Shiva Bashiri, Sharareh Eskandarieh, Mohammad Ali Sahraian, Mansour Rezaei, Negin Fakhri, Kianoosh Khamoushian, Armin Maslehat, Milad Mohamad Yari

**Affiliations:** ^1^Neuroscience Research Center, Health Institute, Kermanshah University of Medical Sciences, Kermanshah, Iran; ^2^Clinical Research Development Center, Imam Reza Hospital, Kermanshah University of Medical Sciences, Kermanshah, Iran; ^3^Multiple Sclerosis Research Center, Neuroscience Institute, Tehran University of Medical Sciences, Tehran, Iran; ^4^Social Development and Health Promotion Research Center, Kermanshah University of Medical Sciences, Kermanshah, Iran; ^5^Neuroscience Research Center, Health Institute, Kermanshah University of Medical Sciences, Kermanshah, Iran; ^6^Clinic of Multiple Sclerosis, Imam Reza Hospital, Kermanshah University of Medical Sciences, Kermanshah, Iran; ^7^Moaven Hospital, Kermanshah University of Medical Sciences, Kermanshah, Iran

**Keywords:** Multiple sclerosis, Environmental risk factors, Lifestyle determinants, Sunlight exposure, Passive smoking

## Abstract

**Background and Objectives:**

Multiple sclerosis (MS) is a chronic immune-mediated disorder of the central nervous system with increasing prevalence in Iran. Identifying modifiable environmental and lifestyle risk factors is essential for disease prevention and public health strategies. Therefore, this study aimed to evaluate environmental, lifestyle, and medical factors associated with multiple sclerosis in Kermanshah, Iran.

**Methods:**

This case-control study was conducted on 300 MS patients and 300 matched healthy controls. Data on demographics, substance use, sun exposure, medical and psychiatric history, family history, and major life stressors were collected using questionnaires. Univariate and multivariable logistic regression analyses were utilized to estimate crude and adjusted odds ratios and 95% confidence intervals.

**Results:**

MS patients were more likely to be female, with lower educational attainment compared to controls. In addition, reduced sun exposure in adolescence and adulthood was strongly associated with MS (*P*<0.05). Moreover, passive smoking during adolescence and maternal smoking during pregnancy were related to higher odds of MS (aOR 1.54, 95% CI: 1.05–2.72 and aOR 3.70, 95% CI: 1.19–11.52, respectively). A history of depression (aOR 3.17, 95% CI: 1.95–5.13) and migraine (aOR 1.94, 95% CI: 1.14–3.30) were also significantly associated with MS. Additionally, a family history of MS in first-degree relatives was more frequent among cases (aOR 2.31, 95% CI: 1.36–3.94). All models were adjusted for gender, ethnicity, and education level.

**Conclusion:**

The findings indicated that MS in Kermanshah is shaped by reduced sunlight exposure, passive and maternal smoking, psychiatric comorbidities, and family history, highlighting several modifiable environmental determinants that may guide targeted prevention efforts and inform public health strategies in high-prevalence regions.

## Background

 Multiple sclerosis (MS) is a chronic immune-mediated inflammatory disorder of the central nervous system that is characterized by focal areas of demyelination, axonal injury, and neurodegeneration within the brain and spinal cord.^1,2^ In addition, it is the leading cause of non-traumatic neurological disability in young adults, with a higher prevalence in women and a typical age of onset between 20 and 50 years.^1,2^ The clinical course of MS varies, ranging from the common relapsing-remitting MS to more progressive phenotypes, including secondary progressive MS, primary progressive MS, and progressive relapsing MS.^1,3,4^

 Globally, an estimated 2–2.5 million individuals are affected by MS, and its prevalence is increasing, particularly in regions with populations of Northern European descent, such as North America and Northern Europe.^2,5^ In contrast, its prevalence remains low in tropical or East Asian regions. However, recent epidemiological data demonstrate that MS is becoming increasingly common in countries like Iran, where it was previously considered rare.^6,7^ Our country is now recognized as a high-risk region, with a national prevalence exceeding 54 cases per 100,000 population and a reported female-to-male ratio ranging from 1.8:1 to 3.6:1.^7,8^ In provinces such as Kermanshah, the burden is even more pronounced, with over 2,400 confirmed cases in a population of around 2 million.

 The etiology of MS is multifactorial, involving a complex interaction between genetic predisposition and environmental triggers.^1,2^ One implicated risk factor includes cigarette smoking, which not only increases MS risk by approximately 50% but may also accelerate conversion from RRMS to progressive forms. Likewise, smoking may promote demyelination through oxidative stress, induction of proinflammatory cytokines, and epigenetic modifications affecting immune tolerance.^9-14^ Moreover, exposure to ultraviolet radiation and low vitamin D levels have been inversely correlated with MS risk. Sunlight may confer protection via vitamin D–dependent and independent immunomodulatory pathways. However, insufficient sunlight exposure can lead to vitamin D deficiency, which may impair immune regulation by reducing anti-inflammatory cytokines while promoting autoreactive T-cell activation. Low serum 25(OH) D levels have been consistently associated with increased MS risk and disease activity.^2,15-17^ Furthermore, multiple studies have shown that stressful life events can trigger MS onset or exacerbate disease progression. Stressful life events and chronic psychological stress can alter hypothalamic–pituitary–adrenal axis activity, resulting in immune dysregulation and heightened inflammatory responses. Overall, these pathways suggest plausible causal links between environmental exposures and the development of MS.^18-23^

 Given the rising incidence of MS in Iran and the vital role of environmental, lifestyle, and psychosocial factors in MS pathogenesis,^24,25^ the present case-control study aims to quantify the associations between these exposures and the occurrence of MS and evaluate whether these relationships persist after adjusting for key sociodemographic confounders, such as gender, ethnicity, and education level. This study is guided by a causal framework in which environmental and lifestyle exposures are considered potential determinants influencing the risk of MS onset, with demographic and socioeconomic variables acting as confounding factors.

## Methods

###  Study Design and Setting

 This case-control study was conducted in Kermanshah Province, Iran, to investigate the association between substance use, sun exposure, medical history, and stressful life events with MS. The study was approved by the Ethics Committee of Kermanshah University of Medical Sciences (ethical code IR.KUMS.MED.REC.1403.060). Then, patients with MS were identified from the National MS Registry of Iran.

###  Sample Size Calculation

 The sample size was estimated based on a two-sided comparison of proportions, assuming a significance level of α = 0.05, statistical power of 80% (β = 0.20), and an expected odds ratio (OR) of approximately 2.0 for key exposures (e.g., low sunlight exposure or passive smoking) based on previous Iranian case-control studies.^13,26^ The minimum required sample size was calculated to be 150 participants per group. To increase the study power and compensate for potential exclusions or incomplete data, the final sample size was expanded to 300 participants in each group.

###  Participants and Matching

 The case group consisted of 300 patients with a confirmed diagnosis of MS according to the 2017 McDonald criteria, identified from the provincial MS registry and neurology clinics. Controls were selected from the same source population as the cases, that is, residents of Kermanshah Province without a history of neurological or autoimmune diseases. A convenience sampling approach was used for this purpose. Healthy volunteers were recruited from public areas (e.g., community centers and workplaces) and among companions or relatives of non-neurological patients attending Imam Reza Hospital, where the MS patients were also identified. It should be noted that all controls were frequency-matched to cases by age (± 2 years) and gender in order to ensure comparability. This approach ensured that both groups represented the same underlying population while minimizing potential selection bias.

###  Inclusion and Exclusion Criteria

 The inclusion criteria for cases were an age range of 18–50 years, a confirmed MS diagnosis by a neurologist according to the 2017 McDonald criteria, residency in Kermanshah for at least the past two years, and a lack of other neurological disorders.

 In addition, the inclusion criteria for controls were the age range of 18–50 years, no history of MS or other central nervous system diseases, and residency in Kermanshah for at least the past two years.

 On the other hand, the exclusion criteria for both groups were inability to provide informed consent, pregnancy, history of severe head trauma with loss of consciousness, and presence of a chronic debilitating disease other than MS in cases.

###  Data Collection

 Data related to environmental, lifestyle, and psychosocial exposures (e.g., sun exposure during adolescence, maternal smoking during pregnancy, and lifetime stressful events) were obtained through self-reported responses using a structured and previously validated questionnaire adapted from the Environmental Risk Factors in MS Questionnaire (EnvIMS-Q), which has been validated and culturally adapted into Persian with established reliability.^25^ Data for both cases and controls were obtained using the same structured and validated questionnaire administered by trained interviewers. For MS patients, demographic and clinical data were extracted from medical records and then verified through structured telephone interviews. It should be noted that telephone interviews were used because some patients lived in remote areas or had mobility limitations that prevented in-person participation.

 Similarly, controls were selected from the general population through convenience sampling and interviewed face-to-face using the same questionnaire and standardized scripts. All interviewers underwent identical training, followed the same data collection protocol, and used the same question wording to minimize interviewer and information bias. Moreover, participants were unaware of the study hypotheses, and exposure questions were framed in a general manner (e.g., “lifetime” or “habitual” behaviors) rather than in relation to disease onset. Finally, all responses were coded and anonymized.

###  Variables and Definitions

 Tobacco exposure included active smoking, passive exposure during childhood/adolescence, and maternal smoking during pregnancy. Sun exposure was separately assessed in adolescence and adulthood. In addition, stressful life events were evaluated using a structured checklist adapted from the Holmes–Rahe Social Readjustment Rating Scale and the Life Events and Difficulties Schedule, previously validated in Persian studies. Participants were asked whether they had experienced bereavement of a close relative, serious illness in a family member, divorce, financial hardship, unemployment, migration, addiction in the family, or other major psychosocial stressors. Each reported event was categorized by severity (major vs. minor) based on its expected impact on daily functioning and by recency (recent: within the past 6 months vs. distant: > 6 months ago). Further, a composite variable of “any major stressful life event” was used in the main analyses. This structured approach ensured comparability with prior MS risk factor studies using the EnvIMS-Q and Life Events and Difficulties Schedule frameworks. Medical history included infections (measles, rubella, mumps, chickenpox, hepatitis B, and mononucleosis) and autoimmune or chronic conditions.

###  Operational Definitions

 To ensure measurement consistency, all key variables were operationally defined as follows:

- Active Smoking: Smoking ≥ 100 cigarettes during lifetime or current daily use at the time of data collection
- Passive Smoking: Regular exposure to tobacco smoke at home or workplace for ≥ 1 hour/day for at least 6 months during adolescence (aged 13–19)
- Maternal Smoking During Pregnancy: Self-reported smoking by the mother while pregnant with the participant
- Sunscreen Use: Regular use defined as applying sunscreen on most days when outdoors during adolescence or adulthood
- Depression: Self-reported physician-diagnosed depression or use of antidepressant medication for ≥ 6 months
- Migraine: Physician-diagnosed migraine headache history according to self-report
- Stressful Life Events: Major events (e.g., bereavement, divorce, severe illness, or financial crisis) occurring within the past 6 months or earlier, as assessed using a structured checklist
- Family History of Multiple Sclerosis: Presence of clinically confirmed MS in first-degree relatives.
- All variables were coded dichotomously (yes/no) or ordinally, as appropriate, and validated through pilot testing for reliability.

###  Sun Exposure Assessment

 Daily sun exposure was assessed using items adapted from the EnvIMS-Q. Participants were asked to estimate their average time spent outdoors on both weekdays and weekends between 10 a.m. and 4 p.m. during adolescence (aged 13–19) and adulthood. Separate questions were asked for summer and winter seasons, including: *“On a typical weekday during summer (and winter), how many hours do you usually spend outdoors between 10 a.m. and 4 p.m.?” “On a typical weekend day during summer (and winter), how many hours do you usually spend outdoors between 10 a.m. and 4 p.m.?”*

 The mean of weekday and weekend values was utilized to calculate average daily sun exposure for each season. Responses were categorized as low (< 1 hour/day), moderate (1–3 hours/day), or high (> 3 hours/day) exposure, consistent with the EnvIMS-Q protocol. Additionally, participants were asked about habitual sunscreen use during adolescence and adulthood (yes/no), defined as regular use on most days spent outdoors.

###  Statistical Analysis

 The obtained data were analyzed using the Statistical Package for the Social Sciences 29.0® (SPSS Inc., Chicago, IL, USA). Continuous variables were expressed as means ± standard deviations and compared using independent *t*-tests or Mann–Whitney *U* tests, depending on normality. Normality of continuous variables was tested using the Kolmogorov–Smirnov test. Moreover, non-normally distributed variables (e.g., daily sun exposure hours) were analyzed using the Mann–Whitney U test, and results are presented as mean ranks. Categorical variables were expressed as frequencies and percentages, which were compared using chi-square or Fisher’s exact tests.

 Univariate (crude) ORs and 95% confidence intervals (CIs) were estimated for each potential risk factor using binary logistic regression. Subsequently, a multivariable logistic regression model was constructed to measure adjusted ORs, controlling for key confounders, including gender, ethnicity (Kurdish vs. non-Kurdish), and education level. Variables with *P* < 0.10 in univariate analyses were entered into the multivariable model. For transparency, ORs with 95% CIs were calculated and reported for all binary exposure variables, regardless of statistical significance, in order to avoid selective reporting bias.

 Although only variables with *P* < 0.10 in univariate analyses were entered into the multivariable model, certain variables, such as gender, ethnicity, and education level, were retained regardless of their *P*-values because they are known confounders supported by prior literature and were part of the study’s a priori causal framework. This approach ensured that effect estimates were adjusted for key demographic covariates and prevented residual confounding.

 For all binary variables, the non-exposed group (e.g., ‘No’ response) was used as the reference category.

 For exposure variables with zero or near-zero frequencies in one or both groups, logistic regression models could not estimate valid ORs due to complete or quasi-complete separation; such results were reported as ‘Not estimable’ in tables.

 A *P*-value < 0.05 was considered statistically significant. Given the number of statistical tests performed, the potential for type I error inflation was taken into account. Therefore, *P*-values were interpreted cautiously, and variables with marginal significance (*P* ≈ 0.05) were further evaluated within multivariable models. Formal correction for multiple comparisons (e.g., Bonferroni adjustment) was not applied, as the analysis was hypothesis-driven and based on predefined risk factors rather than exploratory testing.”

###  Model Diagnostics and Validation

 The assumptions of logistic regression were checked prior to interpreting the final models. Additionally, multicollinearity among independent variables was assessed using the variance inflation factor (VIF). All VIF values were < 2.0, indicating no significant collinearity. Moreover, model fit was evaluated using the Hosmer–Lemeshow goodness-of-fit test (*P* > 0.05 indicating adequate fit), as well as the Nagelkerke R^2^ statistic to assess the explained variance. To estimate model stability, sensitivity analyses were performed by (1) re-running the models after excluding outliers or influential observations (based on standardized residuals > 3.0) and (2) repeating the analysis with and without variables of marginal significance. These checks confirmed the robustness of the main results.

## Results

 In general, 600 participants were included in the analysis, comprising 300 patients with MS and 300 healthy controls matched in terms of age and gender. The mean age of participants was 36.5 ± 2.8 years, and overall, 72.7% were female.

 Beyond these matched factors, significant demographic differences were observed between cases and controls. The proportion of women was higher among MS patients compared with controls (78.3% vs. 67.0%, *P* = 0.005). Based on the results, both paternal and maternal Kurdish ethnicity were more frequent among MS cases than controls (*P* < 0.01). Educational attainment was lower in the MS group, with fewer patients holding a bachelor’s degree or higher, and lower educational levels were also more common among parents of MS patients (*P* < 0.001). In addition, marital status differed modestly, with MS cases less likely to be married than controls (*P* = 0.048), but because multiple statistical tests were conducted, marginal *P*-values (close to 0.05) should be interpreted with caution. Other variables (e.g., dominant hand, hair color, and eye color) demonstrated no significant group differences (Table 1).

**Table 1:** Demographic Characteristics, Educational Attainment, and Natural Hair and Eye Color of Participants

**Variable**	**MS Cases (%)**	**Controls (%)**	***P*** ** value**
Gender (female)	78.3	67.0	0.005
Birth month (June)	13.7	9.3	0.002
Paternal ethnicity (Kurdish)	79.9	65.5	0.001
Maternal ethnicity (Kurdish)	76.0	65.1	0.004
Marriage status (married)	64.2	66.4	0.048
Having a child (yes)	57.7	53.7	0.329
Education level (bachelor’s degree)	36.8	51.8	0.001
Paternal education (low level)	57.5	42.2	0.001
Maternal education (low level)	66.8	50.4	0.001
Dominant hand (right)	90.6	91.6	0.668
Hair color (black)	51.0	47.3	0.288
Eye color (brown)	77.0	69.8	0.247

*Note*. MS: Multiple sclerosis.

 The results (Table 1) confirmed that cases and controls significantly differed in terms of gender, ethnicity, and education level. Therefore, these variables were included as covariates in all multivariable logistic regression models to adjust the reported ORs (Table 2; Table 3 presents sun exposure data; Table 4 reports additional adjusted models.

**Table 2:** Tobacco Use, Illicit Drug Use, Alcohol Consumption, and Other Substance-Related Exposures

**Variables**	**MS Cases**	**Controls**	**Crude OR (95% Cl)**	***P*** **-Value**	**Adjusted OR (95% Cl)**	***P *** **value**
Cigarette smoking, lifetime						
< 180 > 180	261	249	1.00		1.00	
	33	41	0.72 (0.44, 1.19)	0.198	0.94 (0.53, 1.67)	0.824
Cigarette smoking, teenage						
No Yes	287	278	1.00		1.00	
	13	22	0.53 (0.26, 1.10)	0.87	0.64 (0.28, 1.44)	0.281
Current cigarette smoking						
No Yes	281	277	1.00		1.00	
	19	32	0.61 (0.27, 1.39)	0.239	0.91 (0.34, 2.43)	0.852
Hookah use ( ≥ 1 time/week)						
No Yes	255	251	1.00		1.00	
	37	38	1.00 (0.62, 1.62)	0.987	1.07 (0.63, 1.84)	0.799
Living with a smoker at home						
No Yes	157	175	1.00		1.00	
	141	123	1.25 (0.90, 1.73)	0.177	1.02 (0.71, 1.46)	0.925
Living with a smoker at home, teenage						
No Yes	177	227	1.00		1.00	
	121	69	2.21 (1.07, 4.57)	0.032	1.54 (1.05, 2.72)	0.029
Maternal smoking during pregnancy						
No Yes	262	282	1.00		1.00	
	18	5	3.97 (1.46, 10.78)	0.007	3.70 (1.19, 11.52)	0.024
Lifetime illicit drug use						
No Yes	281	287	1.00		1.00	
	16	12	1.36 (0.63, 2.93)	0.430	1.56 (0.65, 3.79)	0.322
Opium use						
No Yes	286	294	1.00		1.00	
	14	6	3.25 (0.43, 24.84)	0.256	3.93 (0.29, 52.99)	0.303
Cannabis use						
No Yes	294	295	1.00		1.00	
	5	4	Not estimable	0.997	Not estimable	0.999
Stimulant use						
No Yes	294	297	1.00		1.00	
	2	0	Not estimable	0.998	Not estimable	0.999
Psychedelic drug use						
No Yes	296	299	1.00		1.00	
	2 (0.7%)	1 (0.3%)	Not estimable	0.999	Not estimable	0.999
Inhalant use						
No Yes	297	298	1.00		1.00	
	1	0	Not estimable	1.000	Not estimable	1.000
Alcohol consumption (any type)						
No Yes	278	262	1.00		1.00	
	20	30	0.60 (0.33, 1.08)	0.089	0.70 (0.36, 1.35)	0.283
Beer consumption						
No Yes	285	275	1.00		1.00	
	10	24	0.40 (0.19, 0.85)	0.018	0.45(0.96, 2.28)	0.057
Wine consumption						
No Yes	286	275	1.00		1.00	
	11	23	0.46 (0.22, 0.97)	0.041	0.55 (0.25, 1.22)	0.142

*Note*. MS: Multiple sclerosis; OR: Odds ratio; CI: Confidence interval. ORs adjusted for gender, ethnicity (Kurdish vs. non-Kurdish), and education level. ORs not estimable due to zero or near-zero cell counts in one or both groups.

**Table 3:** Sun Exposure (Mean Ranks) and Sunscreen Use Frequency by MS Cases and Control Groups

**Variables**	**Season**	**Group**	**Mean Rank/n (%)**	***P*** ** value**
**Daily Sun Exposure (Hours/Day)**				
Aged 13-19 years	Summer	Control	318.15	0.009
		MS cases	281.91	
	Winter	Control	325.84	0.001
		MS cases	272.25	
Aged ≥ 20 years	Summer	Control	316.35	0.019
		MS cases	283.70	
	Winter	Control	328.82	0.001
		MS cases	269.25	
Sunscreen use (never/rarely)				
Aged 13-19 years	-	Control	55.2%	0.002
	-	MS cases	70.0%	
Aged ≥ 20 years	-	Control	37.8%	0.014
	-	MS cases	44.1%	

*Note*. MS: Multiple sclerosis.

**Table 4:** Stressful Life Events, Past Medical History, Antibiotic Use, and Family History of Multiple Sclerosis

**Variables**	**MS Cases**	**Controls**	**Crude OR (95% Cl)**	***P*** **-Value**	**Adjusted OR (95% Cl)**	***P*** ** value**
Death of a first-degree relative						
No Yes	241	251	1.00		1.00	
	56	49	1.19 (0.78, 1.82)	0.419	1.28 (0.80, 2.06)	0.305
History of depression						
No Yes	208	262	1.00		1.00	
	89	30	3.54 (2.26, 5.54)	0.001	3.17 (1.95, 5.13)	0.001
History of rubella						
No Yes	284	274	1.00		1.00	
	16	23	0.86 (0.29, 2.60)	0.793	0.44 (0.12, 1.57)	0.210
History of measles						
No Yes	293	291	1.00		1.00	
	6	7	0.67 (0.34, 1.32)	0.250	0.56 (0.27, 1.18)	0.126
History of mumps						
No Yes	277	269	1.00		1.00	
	20	29	0.66 (0.36, 1.22)	0.185	0.80 (0.42, 1.53)	0.496
History of hepatitis B infection						
No Yes	298	297	1.00		1.00	
	2	3	0.67 (0.11, 4.05)	0.664	1.19 (0.18, 7.01)	0.905
History of chickenpox						
No Yes	208	216	1.00		1.00	
	92	80	1.19 (0.84, 1.70)	0.326	1.22 (0.83, 1.80)	0.307
History of melanoma						
No Yes	298	297	1.00		1.00	
	0	0	Not estimable	-	Not estimable	-
History of Hodgkin lymphoma						
No Yes	299	295	1.00		1.00	
	0	0	Not estimable	-	Not estimable	-
History of head trauma						
No Yes	269	279	1.00		1.00	
	29	17	1.89 (1.01, 3.57)	0.048	1.45 (0.74, 2.87)	0.281
History of migraine						
No Yes	240	269	1.00		1.00	
	56	26	2.39 (1.44, 3.96)	0.001	1.94 (1.14, 3.30)	0.014
History of mononucleosis						
No Yes	293	296	1.00		1.00	
	6	1	3.05 (0.32, 29.50)	0.335	2.81 (0.27, 29.02)	0.385
History of systemic lupus erythematosus						
No Yes	296	297	1.00		1.00	
	2	1	2.03 (0.18, 22.48)	0.565	1.23 (0.10, 15.42)	0.874
History of rheumatoid arthritis						
No Yes	289	290	1.00		1.00	
	8	6	1.36 (0.47, 3.96)	0.577	0.95 (0.30, 2.96)	0.925
History of hypothyroidism						
No Yes	264	261	1.00		1.00	
	28	36	0.76 (0.45, 1.29)	0.303	0.66 (0.37, 1.19)	0.168
History of hyperthyroidism						
No Yes	288	288	1.00		1.00	
	7	9	0.78 (0.29, 2.12)	0.627	0.68 (0.24, 1.94)	0.474
History of type 1 diabetes mellitus						
No Yes	297	297	1.00		1.00	
	2	1	1.01 (0.14, 7.22)	0.992	1.37 (0.11, 16.42)	0.804
History of psoriasis						
No Yes	296	292	1.00		1.00	
	3	5	0.60 (0.14, 2.54)	0.490	1.16 (0.21, 6.27)	0.866
History of urinary tract infection						
No Yes	284	282	1.00		1.00	
	11	14	0.87 (0.38, 1.98)	0.740	0.88 (0.37, 2.09)	0.767
Antibiotic use ≥ 2 weeks in the past 3 years						
No Yes	142	130	1.00		1.00	
	155	165	0.88 (0.63, 1.21)	0.421	1.02 (0.72, 1.46)	0.894
Family history of multiple sclerosis						
No Yes	267	279	1.00		1.00	
	27	16	2.35 (1.43, 3.84)	0.001	2.31 (1.36, 3.94)	0.002

*Note*. MS: Multiple sclerosis; OR: Odds ratio; CI: Confidence interval. ORs adjusted for gender, ethnicity (Kurdish vs. non-Kurdish), and education level. ORs not estimable due to zero or near-zero cell counts in one or both groups.

 Multivariable logistic regression models adjusted for gender, ethnicity, and education level (Table 2) revealed that, regarding lifestyle exposures, no significant differences were observed in overall cigarette smoking or hookah use between cases and controls. However, passive exposure to tobacco was more pronounced among MS patients living with a smoker during adolescence (aged 13–19) (*P* = 0.029, aOR 1.54, 95% CI: 1.05–2.72). Moreover, maternal smoking during pregnancy was considerably higher in MS cases (6.4% vs. 1.7%), corresponding to increased odds of MS (aOR 3.70, 95% CI: 1.19–11.52).

 Likewise, patterns of sun exposure demonstrated notable differences. Considering that sun exposure data were not normally distributed, group comparisons were performed using the non-parametric Mann–Whitney U test, and results are presented as mean ranks (Table 3). Controls reported longer daily sun exposure during both adolescence and adulthood across summer and winter seasons (*P* < 0.05 for all comparisons). Furthermore, sunscreen use was more frequent among controls, while MS patients were significantly more likely to report rare or no sunscreen use during adolescence and adulthood (*P* = 0.002 and *P* = 0.014, respectively, Table 3).

 Based on multivariable logistic regression models adjusted for gender, ethnicity, and education level (Table 4), psychiatric and medical histories revealed important associations. A history of depression was significantly more prevalent among MS patients compared with controls (30% vs. 10.3%; aOR 3.17, 95% CI: 1.95–5.13, *P* < 0.001). Similarly, migraine was more frequently reported in the MS group (18.9% vs. 8.8%; aOR 1.94, 95% CI: 1.14–3.30, *P* = 0.014). Other infectious diseases, autoimmune conditions, and chronic illnesses did not significantly differ between groups. Notably, a positive family history of MS in first-degree relatives was more common in cases (9.2% vs. 5.4%, aOR 2.31, 95% CI: 1.36–3.94, *P* = 0.002). Stressful life events, such as bereavement in first-degree relatives, showed some association (aOR 2.51, 95% CI: 1.61–3.92, *P*< 0.001).

 All final models demonstrated good fit according to the Hosmer–Lemeshow test (*P* > 0.05), with Nagelkerke R^2^ values ranging from 0.18 to 0.27. No evidence of multicollinearity was detected (all VIF < 2.0), and sensitivity analyses yielded consistent results.

## Discussion

 This case-control study investigated demographic, lifestyle, environmental, and medical history factors associated with MS in Kermanshah, Iran. Our findings highlight the multifactorial etiology of MS, demonstrating significant associations with female gender, Kurdish ethnicity, lower educational attainment, reduced sunlight exposure, passive smoking, maternal smoking during pregnancy, psychiatric comorbidities, and family history of MS.

 Consistent with global data, a higher prevalence of MS was observed among women, supporting the established female predominance of the disease.^1,2^ The role of genetic and ethnic background was also evident. Both paternal and maternal Kurdish ethnicity were more frequent among cases, which aligns with prior evidence, suggesting that genetic susceptibility, particularly within certain ethnic groups, contributes to disease risk.^7,8^ Educational level was inversely related to odds of MS, which may reflect socioeconomic determinants, health literacy, or unmeasured lifestyle factors.

 Environmental exposures demonstrated strong associations. Patients with MS reported significantly less sun exposure during adolescence and adulthood, across both summer and winter. This finding reinforces the protective role of ultraviolet radiation and vitamin D pathways in MS pathogenesis.^2,15-17^ Similarly, our results confirmed that sunscreen use was more frequent among controls, indicating that intentional sun avoidance in cases may not explain the observed difference. Overall, these findings conform to those of prior Iranian and international studies linking low sunlight exposure to odds of MS.

 Tobacco-related exposures emerged as notable associated factors. While active smoking was not significantly associated with MS, passive exposure during adolescence was considerably higher in cases. Most strikingly, maternal smoking during pregnancy increased MS odds more than threefold, which is consistent with earlier evidence that early-life tobacco exposure influences immune development and disease susceptibility.^10-14^ These findings emphasize the importance of considering both direct and indirect smoking exposures in MS epidemiology.

 Psychiatric and neurological comorbidities were prominent. A history of depression was four times more frequent among MS patients, which is in line with the findings of previous studies, demonstrating the bidirectional relationship between MS and depression. ^18-20^ Additionally, migraine showed a strong association, echoing prior reports of shared vascular and inflammatory pathways between migraine and MS.^21,22^ Infectious mononucleosis was rare but more common among cases, supporting the growing body of evidence linking Epstein–Barr virus infection to MS onset.^1,2,9,26^

 Finally, stressful life events (e.g., bereavement) were more frequently reported among MS patients, which corroborates the results of previous studies, identifying psychosocial stressors as potential triggers for disease onset or relapse.^18-23^ Although stress within the preceding six months was not different between groups, lifetime exposure appears to play a more critical role. In addition, a family history of MS among first-degree relatives was more common among patients, highlighting the interplay between genetic susceptibility and environmental triggers.^7,8^

 The associations observed in this study are biologically plausible and supported by several proposed mechanisms in MS pathogenesis. Low sun exposure during adolescence may reduce endogenous vitamin D synthesis, leading to impaired immunoregulation, decreased anti-inflammatory cytokine production, and enhanced autoreactive T-cell activation. Furthermore, smoking-related associations may be mediated through oxidative stress, increased pro-inflammatory cytokines, and epigenetic modifications affecting immune tolerance. The link between stressful life events and MS may be explained by the dysregulation of the hypothalamic-pituitary-adrenal axis, resulting in altered cortisol dynamics and subsequent immune imbalance. Additionally, early-life exposures (e.g., maternal smoking) may influence fetal immune development through inflammatory and toxic pathways. In general, these mechanisms provide biological support for the associations identified in this study.

 The strengths of this study included its relatively large sample size, rigorous diagnostic criteria, and inclusion of diverse environmental and lifestyle exposures. Importantly, groups were matched only for age and gender, allowing demographic variables (e.g., ethnicity and education) to be analyzed as independent associated factors. Nevertheless, limitations should be acknowledged. Recall bias may have influenced self-reported exposures, particularly for stressful life events and substance use. Moreover, alcohol and illicit drug use were likely underreported due to cultural and legal constraints in Iran. Another limitation of this study was the evaluation of infectious mononucleosis history based on participant interviews. Given the nonspecific symptoms of this infection and the possibility that many individuals may not recall or recognize a past episode, recall bias is likely. This limitation may have affected the accuracy of our estimates regarding the association between infectious mononucleosis and MS.

 Although identical questionnaires and interviewer training were applied, the use of telephone interviews for MS cases and face-to-face interviews for controls might have introduced minor information bias. This approach, however, was necessary due to the mobility limitations and geographical dispersion of MS patients. In addition, as several exposure variables (e.g., sun exposure, maternal smoking, and lifetime stress) were self-reported, recall bias cannot be completely excluded. Nevertheless, identical questionnaires and interviewer training were used for both groups in order to reduce differential misclassification.

 Reverse causation is a potential concern in interpreting the observed association between depression and MS. Although participants were asked to report depressive symptoms or diagnoses prior to MS onset, it is possible that prodromal or early neurological changes related to MS may have contributed to the development of depressive symptoms. Therefore, the association between depression and MS onset should be interpreted with caution, as depression may partly reflect early disease manifestations rather than an independent antecedent risk factor.

 Several associations in this study were accompanied by wide 95% CIs, particularly for exposures with low prevalence, such as maternal smoking during pregnancy, infectious mononucleosis, and other rare lifestyle or medical factors. These wide intervals indicate reduced precision of the corresponding estimates and are likely the result of limited statistical power for low-frequency variables, even with a relatively large sample size of 300 cases and 300 controls. Accordingly, such effect estimates should be interpreted with caution.

 Finally, given the number of statistical tests performed in this study, there is a possibility of false-positive results due to multiple comparisons. However, the key variables analyzed in this investigation were predefined based on existing literature, and multivariable analyses were utilized to confirm independent associations.

**Highlights**

- Reduced sun exposure was linked to higher multiple sclerosis (MS) risk in Kermanshah, Iran.
- Maternal smoking during pregnancy tripled the risk of MS.
- Depression and migraine were strongly associated with MS prevalence.
- Kurdish ethnicity and low education increased susceptibility to MS.
- The findings highlight modifiable risk factors for targeted prevention.

## Conclusion

 Overall, our findings confirmed that MS in Kermanshah is influenced by both established and region-specific associated factors, including reduced sunlight exposure, passive smoking, maternal smoking during pregnancy, depression, migraine, and stressful life events. These findings underscore the multifactorial nature of MS and modifiable associated factors that may inform prevention strategies and targeted public health interventions in high-prevalence regions.

## Acknowledgments

 We would like to acknowledge the use of Grammarly genAI to improve this article through editing the English language and checking the spelling, grammar, and syntax.

## Artificial Intelligence Use Statement

 Artificial intelligence tools were not used for data collection, analysis, or interpretation in this study. However, Grammarly’s AI-based language editing tool was utilized solely for minor English grammar, spelling, and syntax corrections after the manuscript was drafted. Moreover, no generative AI system was involved in conceptualization, statistical analysis, or result interpretation.

## Competing Interests

 The authors declared no conflict of interests.

## Ethical Approval

 This study was approved by the Ethics Committee of Kermanshah University of Medical Sciences (ethical code IR.KUMS.MED.REC.1403.060). In addition, oral informed consent was obtained from all participants prior to enrollment. Further, all collected data were anonymized, coded, and analyzed confidentially to ensure privacy.

## Funding

 This study was extracted from a thesis and supported by Kermanshah University of Medical Sciences, Kermanshah, Iran.
